# Radiation Oncologists’ Perceptions of Adopting an Artificial Intelligence–Assisted Contouring Technology: Model Development and Questionnaire Study

**DOI:** 10.2196/27122

**Published:** 2021-09-30

**Authors:** Huiwen Zhai, Xin Yang, Jiaolong Xue, Christopher Lavender, Tiantian Ye, Ji-Bin Li, Lanyang Xu, Li Lin, Weiwei Cao, Ying Sun

**Affiliations:** 1 Office of Research Management and Education Administration Sun Yat-sen University Cancer Center, State Key Laboratory of Oncology in South China Collaborative Innovation Center for Cancer Medicine Guangzhou China; 2 Department of Anthropology School of Sociology and Anthropology Sun Yat-sen University Guangzhou China; 3 Department of Radiation Oncology Sun Yat-sen University Cancer Center, State Key Laboratory of Oncology in South China Collaborative Innovation Center for Cancer Medicine Guangzhou China; 4 School of Management Sun Yat-sen University Guangzhou China; 5 School of Management Guangdong Ocean University Zhanjiang China; 6 School of Medicine Southern University of Science and Technology Shenzhen China; 7 Department of Clinical Research Sun Yat-sen University Cancer Center, State Key Laboratory of Oncology in South China Collaborative Innovation Center for Cancer Medicine Guangzhou China; 8 Management Office of Huangpu Campus Sun Yat-sen University Cancer Center, State Key Laboratory of Oncology in South China Collaborative Innovation Center for Cancer Medicine Guangzhou China

**Keywords:** artificial intelligence, technology acceptance model, intension, resistance

## Abstract

**Background:**

An artificial intelligence (AI)–assisted contouring system benefits radiation oncologists by saving time and improving treatment accuracy. Yet, there is much hope and fear surrounding such technologies, and this fear can manifest as resistance from health care professionals, which can lead to the failure of AI projects.

**Objective:**

The objective of this study was to develop and test a model for investigating the factors that drive radiation oncologists’ acceptance of AI contouring technology in a Chinese context.

**Methods:**

A model of AI-assisted contouring technology acceptance was developed based on the Unified Theory of Acceptance and Use of Technology (UTAUT) model by adding the variables of perceived risk and resistance that were proposed in this study. The model included 8 constructs with 29 questionnaire items. A total of 307 respondents completed the questionnaires. Structural equation modeling was conducted to evaluate the model’s path effects, significance, and fitness.

**Results:**

The overall fitness indices for the model were evaluated and showed that the model was a good fit to the data. Behavioral intention was significantly affected by performance expectancy (*β*=.155; *P*=.01), social influence (*β*=.365; *P*<.001), and facilitating conditions (*β*=.459; *P*<.001). Effort expectancy (*β*=.055; *P*=.45), perceived risk (*β*=−.048; *P*=.35), and resistance bias (*β*=−.020; *P*=.63) did not significantly affect behavioral intention.

**Conclusions:**

The physicians’ overall perceptions of an AI-assisted technology for radiation contouring were high. Technology resistance among Chinese radiation oncologists was low and not related to behavioral intention. Not all of the factors in the Venkatesh UTAUT model applied to AI technology adoption among physicians in a Chinese context.

## Introduction

### Background

The artificial intelligence (AI) race has long since begun; its silicone roots date back to 1956 [1]. In China’s “Development Plan for The New Generation of Artificial Intelligence,” the goal was to synchronize China’s overall technology and AI applications with the world's advanced level of technology by 2020. The total value of China’s AI industry market is expected to reach US $127 billion by 2025 [2], and the medical AI industry is forecasted to account for one-fifth of the overall AI market [3]. In the health care industry, AI has been developed for administrative assistance, clinical decision-making support, patient monitoring, and robotic surgeries [4]. In clinical decision-making support, computer software is used to analyze clinical data and assist medical professionals in making more precise decisions on disease diagnoses and treatments [5]. Sketching tumors is one such subfield.

Delineating tumors is tricky and requires much education, guidance, and expertise. For radiation oncologists, the tumor target contouring process for precision head and neck radiation therapy is time consuming. It requires, on average, 180 minutes of intense work for each patient, and contouring areas that are created by different radiation oncologists for the same tumor can be highly variable [6]. Moreover, contouring accuracy is key for achieving successful treatment outcomes. Suboptimal tumor coverage and poor-quality radiation therapy plans are major factors of disease relapse and inferior survival [7]. An AI contouring tool that automates the primary gross tumor volume contouring process for patients with cancer was developed at Sun Yat-sen University Cancer Center, wherein it has been applied clinically since 2019; over 12,500 cases of radiation planning have been carried out with the tool’s assistance. The contouring system has a high level of accuracy (ie, when compared with the manual contouring accuracy of radiation oncologists). Although oncologists still need to edit automated contours by making minor adjustments, the system substantially saves time by up to 39.4%, and intraobserver and interobserver variations have been reduced by 36.4% and 54.5%, respectively [8].

Although the benefits of AI assistance appear to be obvious, like all forms of change, the adoption of AI technology might be met with resistance, especially within health care settings; it has been said that the only constant is change [9]. There is much hope and fear surrounding such technologies, and this fear can manifest as resistance from both health care professionals and patients [10]. One particular fear that may promote resistance is the fear that AI tools will replace workers (even skilled workers like doctors)—the much discredited, but still prevalent, Luddite fallacy [11]. In a *British Medical Journal* head-to-head debate, the topic was as follows: “Could machines using artificial intelligence make doctors obsolete?” Dr Jörg Goldhahn said “yes” and foreboded that humans simply cannot keep up with the data crunching advantage that computers have over humans [12]. Further, Vanessa Ramptom and Professor Giatgen Spinas claimed that the role of doctors will not become obsolete; instead, these roles will change to specifically focus on treating a patient as a whole [12]. These perspectives encompass part of the larger narratives that describe AI technology as either utopian or dystopian.

There are also claims that doctors are slow to uptake new technologies. In 2004, there was evidence of resistance across the United States against using a computerized order entry system to overcome issues, such as doctors’ illegible handwriting, and reduce the number of incorrect prescriptions. The Cedars-Sinai Medical Center notably forced the withdrawal of the system, which was installed in two-thirds of the 870-bed hospital [13]. Similarly, in 2006, Bhattacherjee and Hikmet [14,15] found that physicians were resistant to adopting administrative health care information technologies, and more recently, in 2014, Hsieh [16] discovered 5 factors (attitude, subjective norm, perceived behavior control, institutional trust, and perceived risk) that influenced the adoption of such technologies. Notably, these studies all concerned administrative technologies. There is, however, also evidence of doctors resisting AI technology for other uses, such as patient diagnosis and treatment plan development.

Even though the National Institute of Health claims that AI technology is trending in cancer care [17], a number of notable AI projects have failed in the United States. The MD Anderson Cancer Center, one of the top cancer centers in the world, cancelled a project that involved IBM Corporation’s oncology expert advisor tool, Watson, in 2016 after spending US $62 million [18]. In February 2021, IBM Corporation also tried to sell their Watson Health business, as it was not profitable [19].

In this study, we assessed whether there has been any resistance to the uptake of an AI contouring system in China and examined the system’s pros and cons. Few prior studies have focused on the technology acceptance of AI tools that provide clinical decision-making support in cancer care from physicians’ perspectives. AI health care researchers have primarily analyzed technical innovation and clinical results but have seldom considered the human contexts that are invariably involved in use behavior. Social and implemental issues need to be addressed when an AI tool is introduced in hospitals. Further, it is especially important to examine the extent of perceived risks and technology resistance bias.

### Objective

The objective of this study was to develop and test a model for investigating the factors that drive Chinese radiation oncologists’ acceptance of an AI contouring tool for automating the primary gross tumor volume and normal tissue contouring process. This study analyzes how expectancy, social influence, facilitating conditions, perceived risk, and resistance bias contribute to the Technology Acceptance Model (TAM).

### Theoretical Background and Hypothesis Development

The theoretical models in this study included the TAM and the Unified Theory of Acceptance and Use of Technology (UTAUT) model. The perceived risk and status quo bias theory variables were added to the UTAUT model.

The first TAM was developed by Fred D Davis in 1989, and it was based on the theory of reasoned action. The TAM was used to analyze consumers’ behaviors in the context of health information technology based on the following two major variables: perceived usefulness and perceived ease of use [20]. Later, in 2000, Venkatesh and Davis [21] developed the TAM2, which also addressed perceived usefulness and use intentions in terms of social influence and cognitive instrumental processes. They added the subjective norm, voluntariness, image, job relevance, output quality, and result demonstrability constructs, which significantly influenced user acceptance. The evolution of the TAM3 came next; the TAM3 was a comprehensive nomological network (ie, an integrated model) for evaluating individual-level (information technology) adoption and use [22].

In 2003, Venkatesh et al [23] also created a UTAUT model, which is an integrated tool based on 8 original models that were published in major information systems journals from a range of disciplines. These models included the theory of reasoned action model, the TAM, the motivation model, the theory of planned behavior model, a combination of the TAM and the theory of planned behavior model, the model of PC utilization, the innovation diffusion theory model, and the social cognitive theory model. The UTAUT originally explained technology acceptance among employees, and in 2012, a UTAUT2 model was developed, which focused on consumer technologies [24]. As AI-assisted contouring technology has been developed for medical staff in hospitals, hedonic motivation and price value, which were explored in the UTAUT2 model, were considered inapplicable to explaining oncologists’ acceptance behaviors in this study. Therefore, we used the original UTAUT model in this study. The original model and its various modifications have been popularly used in studies of technology adoption in the health care industry over the past 5 years [25-28].

The UTAUT model revealed the following four variables: performance expectancy, effort expectancy, social influence (which is used to evaluate the behavioral intention to use a new technology), and facilitating conditions (which are used to evaluate use behavior). Most research has proven that these four constructs positively affect potential users’ behavioral intentions [29,30]. Performance expectancy is defined as “the degree to which an individual believes that using a new technology will help him or her to attain gains in job performance.” This construct was integrated from a similar concept in the TAM—perceived usefulness [31]. Effort expectancy is defined as “the degree of ease of use associated with IT” [23]. This concept is similar to the perceived ease of use construct in the TAM and innovation diffusion theory and the complexity of technology construct in the model of PC utilization. Social influence is defined as “the degree to which an individual perceives the importance of how others believe that he or she should use new IT.” This concept was developed based on the notion that “an individual’s behavior is influenced by the way in which one believes others will view him/her as a result of having used health IT” [23]. The Venkatesh model integrated subjective norms from the theory of reasoned action, the TAM2, and the theory of planned behavior; social factors from the model of PC utilization; and the image construct from the innovation diffusion theory into the social influence construct. The facilitating conditions construct is defined as “the degree to which an individual believes that an organizational and technical infrastructure exists to support the use of the system” [23]. The UTAUT’s facilitating conditions concept contains the perceived behavior control concept from the theory of planned behavior and the combined TAM and theory of planned behavior model, the facilitating conditions concept from the model of PC utilization, and the compatibility construct from the innovation diffusion theory [23]. Lastly, the intention-usage behavior relationship has long been discussed in many studies of technology acceptance, and we expected that behavioral intentions to use AI-assisted technology would have a significant influence on use behavior. Based on these variables, we proposed the following hypotheses:

- Hypothesis 1: Performance expectancy positively affects oncologists’ behavioral intentions to use an AI contouring tool for developing radiation therapy plans.
- Hypothesis 2: Effort expectancy positively affects oncologists’ behavioral intentions to use an AI contouring tool for developing radiation therapy plans.
- Hypothesis 3: Social influence positively affects oncologists’ behavioral intentions to use an AI contouring tool for developing radiation therapy plans.
- Hypothesis 4: Oncologists’ behavioral intentions positively affect their use behaviors when using an AI contouring tool for developing radiation therapy plans.
- Hypothesis 5A: Facilitating conditions positively affect oncologists’ use behavior when using an AI contouring tool for developing radiation therapy plans.
- Hypothesis 5B: Facilitating conditions positively affect oncologists’ behavioral intentions to use an AI contouring tool for developing radiation therapy plans.

Perceived risk has long been discussed as a negative factor in health care information technology adoption studies [10,16], and it refers to the combination of uncertainty and the seriousness of an outcome [32]. Featherman and Pavlou [33] identified the following seven facets of e-service–related perceived risk: performance risk, financial risk, time risk, physiological risk, social risk, privacy risk, and overall risk. In this study, we addressed perceived risk based on the following four aspects: (1) performance risk, which we defined as the probability of malfunction and performance failure and whether the AI contouring tool could mislead physicians with inaccurate contouring; (2) time risk, which we defined as the probability of physicians needing more time to correct errors caused by the AI system; (3) psychological risk, which we analyzed in terms of whether using the AI contouring tool may cause psychological discomfort due to having a negative effect on clinicians’ self-perceptions of the treatment plan; and (4) privacy risk, which we defined as a potential loss of the confidential patient data in the AI-assisted system. As the AI systems were purchased by public hospitals without input from individual physicians, no financial or social risk assessment was required.

Resistance to change refers to people’s attempts to maintain previous behaviors or habits that are connected to their past experiences when facing changes [15,34,35], and this has been discussed as a major barrier to health care information technology adoption [15,36]. The fear of job loss has also been discussed as a factor of the slow adoption of new information technologies [37]. We suggest that radiation oncologists might believe that AI-assisted technologies might replace them and that the adoption of such technologies will lead to an increase in unemployment rates. Thus, we included these two factors in the resistance bias construct, which we defined as people’s resistance to using a new technology resulting from their resistance to change and concerns about unemployment. Therefore, we proposed the following hypotheses:

- Hypothesis 6: Perceived risk negatively affects oncologists’ behavioral intentions to use an AI contouring tool for developing radiation therapy plans.
- Hypothesis 7: Resistance bias negatively affects oncologists’ behavioral intentions to use an AI contouring tool for developing radiation therapy plans.

## Methods

### Participants and Sampling

The majority of participants were radiation oncologists (227/307, 73.9%) and medical students (80/307, 26.1%) from the Department of Radiation Oncology at Sun Yat-sen University Cancer Center who had clinical experience in using the computational system for contouring. The sample pool also included radiation oncologists from other hospitals in China. Participants were recruited between July 2020 and November 2020. A convenience sampling method was used in this study, as only a few hospitals in China have AI-assisted contouring systems. As our survey had 29 items, the required number of participants was more than 290. The questionnaire was conducted by sending direct website links via email or WeChat (a social media platform developed by Tencent Holding Limited). Questionnaires were only considered valid if (1) an account responded only once and (2) the total response time for completing the questionnaire was more than 60 seconds. In total, 322 radiation oncologists replied to the web-based survey. After filtering out the invalid responses, 307 responses—176 from Sun Yat-sen University Cancer Center and 131 from other hospitals—were deemed valid for data analysis.

### Measurements

Our model included 8 constructs with 29 questionnaire items. Each item assessed 1 construct and was sourced from relevant literature related to new technology acceptance research. Some changes were made to fit the AI-assisted contouring system context (Table 1). After we developed a list of questions, a team discussion was held to reach a consensus. Afterward, we asked a group of 10 oncologists with experience in working with the AI system to answer the questions and provide comments for system improvement. A 7-point Likert scale, which ranged from 1 (strongly disagree) to 7 (strongly agree), was used to assess the items for all constructs except those for the use behavior construct, in which the answers were “yes” or “no.”

The first page of the questionnaire provided an overview of this study’s background, purpose, voluntary nature, and anonymity and an introduction of the AI-assisted tumor volume contouring system, which included information on its general functions and operating procedures and images of the system to help the participants gain a clear understanding. We developed 2 questionnaires to assess oncologists’ perceptions. All participants first answered the following question: “Have you used the AI-assisted contouring system?” If the answer was “yes,” then they were directed to answer the questions for those who have already used the system (Table 1); if the answer was “no,” then they were directed to answer the questions for those who have not used the system. The questionnaires were identical apart from the usage of verb tenses. The questions were translated from English to Mandarin Chinese for the survey. Ethical approval was obtained from the Ethics Committee of the Department of Anthropology at Sun Yat-sen University.

**Table 1:** Questionnaire items related to the research model constructs.

Variables and items		Questionnaire items	Sources
**PE^a,b^**			
	PE1	I find the automated contouring system useful in my job.^c^ I think the automated contouring system will be useful in my job.^d^	Venkatesh et al [23] and Kijsanayotin et al [25]
	PE2	Using the automated contouring system enables me to accomplish tasks quicker.^c^ I think using the automated contouring system will enable me to accomplish tasks quicker.^d^	Venkatesh et al [23] and Kijsanayotin et al [25]
	PE3	Using the automated contouring system increases my productivity.^c^ I think using the automated contouring system will increase my productivity.^d^	Venkatesh et al [23] and Kijsanayotin et al [25]
	PE4	Using the automated contouring system improves the outcomes of my work.^c^ I think using the automated contouring system will improve the outcomes of my work.^d^	Venkatesh et al [23] and Kijsanayotin et al [25]
**EE^a,e^**			
	EE1	The automated contouring system is clear and understandable to me.^c^ I think the automated contouring system will be clear and understandable to me.^d^	Venkatesh et al [23] and Kijsanayotin et al [25]
	EE2	It was easy for me to become skillful at using the automated contouring system.^c^ I think it will be easy for me to become skillful at using the automated contouring system.^d^	Venkatesh et al [23] and Kijsanayotin et al [25]
	EE3	I find the automated contouring system easy to use.^c^ I think the automated contouring system will be easy to use.^d^	Venkatesh et al [23] and Kijsanayotin et al [25]
	EE4	Learning to operate the automated contouring system is easy for me.^c^ I think learning to operate the automated contouring system will be easy for me.^d^	Venkatesh et al [23] and Kijsanayotin et al [25]
**SI^a,f^**			
	SI1	People (eg, colleagues and friends) who influence my behavior think that I should use the automated contouring system.^c,d^	Venkatesh et al [23] and Kijsanayotin et al [25]
	SI2	People who are important to me (eg, department heads, tutors, superiors, and hospital leaders) think that I should use the automated contouring system.^c,d^	Venkatesh et al [23] and Kijsanayotin et al [25]
	SI3	Social propaganda has been helpful in the use of the automated contouring system.^c,d^	Venkatesh et al [23] and Kijsanayotin et al [25]
	SI4	In general, my hospital and my department have supported the use of the automated contouring system.^c,d^	Venkatesh et al [23] and Kijsanayotin et al [25]
**FC^a,g^**			
	FC1	I have the resources (devices, support from colleagues, etc) necessary to use the automated contouring system.^c,d^	Venkatesh et al [23] and Kijsanayotin et al [25]
	FC2	I have the knowledge (the clinical and computer skills) necessary to use the automated contouring system.^c,d^	Venkatesh et al [23] and Kijsanayotin et al [25]
	FC3	Health and information technology personnel in the hospital are available to assist with system difficulties.^c,d^	Venkatesh et al [23] and Kijsanayotin et al [25]
	FC4	I have adequate knowledge resources (eg, books, documents, and consultants) to help me learn about the automated contouring system.^c,d^	Venkatesh et al [23] and Kijsanayotin et al [25]
**BI^a,h^**			
	BI1	I intend to use the automated contouring system in the next 2 months.^c,d^	Venkatesh et al [23] and Kijsanayotin et al [25]
	BI2	I will use the automated contouring system in the next 2 months.^c,d^	Venkatesh et al [23] and Kijsanayotin et al [25]
	BI3	I plan to use the automated contouring system in the next 2 months.^c,d^	Venkatesh et al [23] and Kijsanayotin et al [25]
**PR^a,i^**			
	PR1	There is a possibility of malfunction and performance failure, so the system might fail to deliver accurate contouring areas and could mislead my work with inaccurate contouring.^c,d^	Ye et al [10], Bhattacherjee and Hikmet [15], and Andrews et al [38]
	PR2	There is a probability that I need more time to fix the errors and nuances of the artificial intelligence system.^c,d^	Ye et al [10], Bhattacherjee and Hikmet [15], and Andrews et al [38]
	PR3	I think using the automated contouring system may cause psychological distress, as it could have a negative effect on my self-perception of the treatment plan.^c,d^	Ye et al [10], Bhattacherjee and Hikmet [15], and Andrews et al [38]
	PR4	I am concerned that my patients’ personal information and health details are/would be insecure and could be accessed by stakeholders or unauthorized persons, leading to lawsuits for physicians and the hospital.^c,d^	Ye et al [10], Bhattacherjee and Hikmet [15], and Andrews et al [38]
**RB^a,j^**			
	RB1	I do not want the automated contouring system to change how I develop my treatment plan for manual contouring because the new system is unfamiliar to me.^c,d^	Bhattacherjee and Hikmet [15] and Dou et al [34]
	RB2	I do not want to use the automated contouring system because of past experience; these new high-tech products always fall flat during practical application.^c,d^	Bhattacherjee and Hikmet [15] and Dou et al [34]
	RB3	I do not want to use the automated contouring system because there is a possibility of losing my job, as artificial intelligence–assisted technology may do my work better than me.^c,d^	Raza et al [37]
**UB^k,l^**			
	UB1	I have already used the automated contouring system.^c^ I am preparing to use the automated contouring system.^d^	Venkatesh et al [23]
	UB2	I recommend that others should use the automated contouring system.^c,d^	Venkatesh et al [23]
	UB3	Have you ever overridden the contouring system after using it for some time?^c^ Would you ever override the contouring system?^b^	Venkatesh et al [23]

^a^7-point scale (1=strongly disagree; 2=quite disagree; 3=slightly disagree; 4=neither agree or disagree; 5=slightly agree; 6=quite agree; 7=strongly agree).

^b^PE: performance expectancy.

^c^Questions for those who have already used the system.

^d^Questions for those who have not used the system.

^e^EE: effort expectancy.

^f^SI: social influence.

^g^FC: facilitating conditions.

^h^BI: behavioral intention.

^i^PR: perceived risk.

^j^RB: resistance bias.

^k^2-point scale (1=yes; 2=no).

^l^UB: use behavior.

### Data Analysis

**Figure 1:**
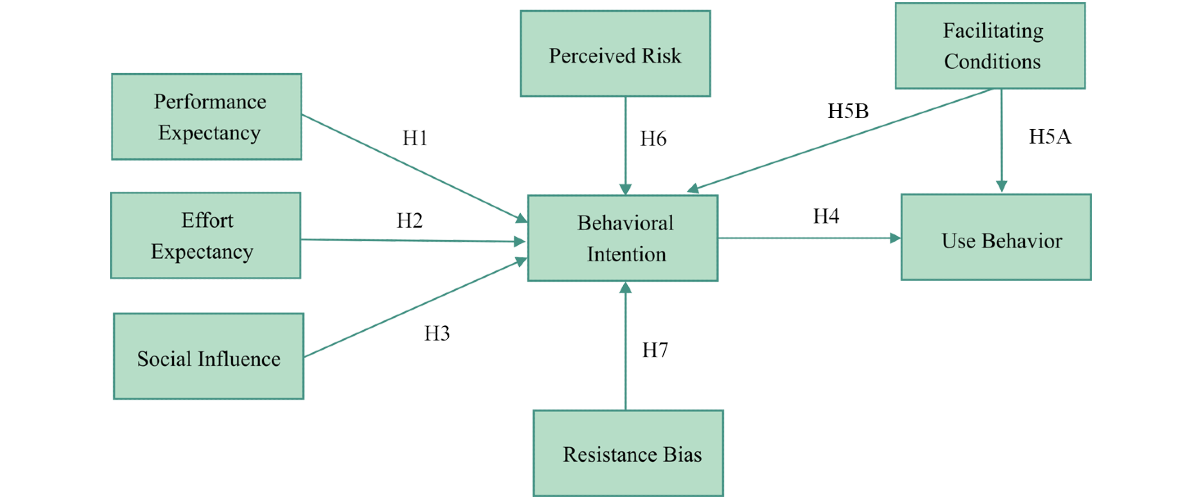
The development of our model for investigating radiation oncologists' acceptance of an artificial intelligence–assisted contouring system. H: hypothesis.

Structural equation modeling analysis was conducted by using IBM SPSS Amos 21.0 (IBM Corporation), and this was a 2-step analysis [39]. First, items and constructs were evaluated for reliability and validity via a confirmatory factor analysis of the measurement model. Second, the structural model’s path effects, significance, and goodness of fit and mediation effects were evaluated. More specifically, the path analysis was performed by conducting a series of multiple regression analyses to derive the standardized regression coefficients (ie, *β*) for all proposed paths. Figure 1 shows the conceptual model for the analysis; the arrows with solid lines are the paths indicating the relationships among the specified concepts in the hypothesis. IBM SPSS Version 25.0 (IBM Corporation) was used for the descriptive statistics analysis.

## Results

### Demographic Results

The demographic characteristics of the 307 participants are shown in Table 2. About an equal number of men (151/307, 49.2%) and women (156/307, 50.8%) participated in this study. The majority (269/307, 87.6%) of the participants’ ages ranged between 18 and 40 years, and all of the participants had at least a bachelor’s degree. In China, radiation oncologists who work on contouring are often aged under 40 years, as senior oncologists mainly focus on checking the accuracy of contouring based on the treatment plans, which are mostly developed by junior oncologists. Thus, it was unsurprising that we had more participants aged under 40 years. In our study, 185 participants had at least 4 years of experience in radiation oncology, 123 participants had already used the AI-assisted contouring technology, and 184 had not yet used it.

**Table 2:** Demographic characteristics (N=307).

Characteristics		Participants, n (%)
**Sex**		
	Male	151 (49.2)
	Female	156 (50.8)
**Age (years)**		
	18-25	121 (39.4)
	26-30	67 (21.8)
	31-40	81 (26.4)
	41-50	30 (9.8)
	>50	8 (2.6)
**Education**		
	Bachelor’s degree	137 (44.6)
	Master’s degree or doctoral degree	170 (55.4)
**Experience in radiation oncology (years)**		
	≤3	122 (39.7)
	4-6	61 (19.9)
	7-10	23 (7.5)
	>10	101 (32.9)
**Artificial intelligence–assisted contouring technology usage**		
	Yes	123 (40.1)
	No	184 (59.9)

### Measurement Model

To test the reliability and validity of this study, we examined common method bias, as all items in our survey were collected from the same questionnaire. We connected all of the items to a common latent factor and used a confirmatory factor analysis to conduct a single-factor test [40]. The results showed that the fit of the single-factor model was poor (*χ*^2^_377_=4294.616; *χ*^2^/df=11.392; adjusted goodness of fit index=0.354; comparative fit index=0.628; Tucker–Lewis index=0.599; root mean square error of approximation=0.184), indicating that common method bias was insignificant in this study.

We also conducted some tests to examine the constructs’ reliability, and the results are summarized in Table 3. The Cronbach *α* and composite reliability values were higher than the threshold of 0.7 [41], which indicated good construct reliability. For convergent validity, all item loadings ranged from 0.633 to 0.980, and good composite reliability (>0.7) was achieved [42].

Convergent validity was assessed with average variance extracted (AVE) values; all construct AVE values were greater than 0.5 [43] and thus showed good convergent validity. In Table 4, the square roots of the AVE values were higher than the numbers in the off-diagonal direction (correlations between a particular construct in the same column and other constructs in different rows) in the corresponding columns, indicating the good discrimination validity of all constructs. The results of a single-factor analysis of variance showed that the main effect that experience had on behavioral intention was not significant (*F*_3,303_=2.587; *P*=.053).

The mean scores for behavioral intention (5.484 out of 7.0) and use behavior (1.713 out of 2.0) were high, which showed that the participants had high intentions to use the AI-assisted contouring system. However, even though the total degree of resistance was low, 17.6% (54/307) of the respondents showed a degree of resistance (ranked as an average score of ≥5 for resistance bias). Specifically, 13 out of the 123 (10.6%) participants who had accessed the AI system showed a degree of resistance, and 41 out of the 184 (22.3%) participants who had not accessed the AI system also showed a degree of resistance.

**Table 3:** Descriptive statistics of variables, items, composite reliability, and convergent validity.

Variable and item		Item loadings	Mean score of the items	Composite reliability	Convergence validity	Cronbach *α*
**PE^a^**			5.969	0.976	0.910	.976
	PE1	0.946				
	PE2	0.970				
	PE3	0.957				
	PE4	0.942				
**EE^b^**			5.669	0.970	0.891	.970
	EE1	0.921				
	EE2	0.933				
	EE3	0.967				
	EE4	0.954				
**SI^c^**			5.481	0.938	0.790	.937
	SI1	0.909				
	SI2	0.906				
	SI3	0.829				
	SI4	0.909				
**PR^d^**			4.418	0.826	0.544	.824
	PR1	0.633				
	PR2	0.735				
	PR3	0.783				
	PR4	0.788				
**RB^e^**			3.528	0.925	0.805	.825
	RB1	0.865				
	RB2	0.941				
	RB3	0.883				
**FC^f^**			5.446	0.929	0.766	.926
	FC1	0.821				
	FC2	0.886				
	FC3	0.899				
	FC4	0.892				
**BI^g^**			5.484	0.980	0.942	.980
	BI1	0.952				
	BI2	0.980				
	BI3	0.979				
**UB^h^**			1.713	0.820	0.604	.800
	UB1	0.736				
	UB2	0.860				
	UB3	0.729				

^a^PE: performance expectancy.

^b^EE: effort expectancy.

^c^SI: social influence.

^d^PR: perceived risk.

^e^RB: resistance bias.

^f^FC: facilitating conditions.

^g^BI: behavioral intention.

^h^UB: use behavior.

**Table 4:** Discriminant validity of the constructs. The square roots of the average variance extracted values for comparisons among constructs are shown.^a^

Constructs	Performance expectancy	Effort expectancy	Social influence	Facilitating conditions	Perceived risk	Resistance bias	Behavioral intention	Use behavior
Performance expectancy	0.954	—^b^	—	—	—	—	—	—
Effort expectancy	0.767	0.944	—	—	—	—	—	—
Social influence	0.717	0.784	0.889	—	—	—	—	—
Facilitating conditions	0.665	0.712	0.716	0.875	—	—	—	—
Perceived risk	−0.334	−0.378	−0.358	−0.314	0.738	—	—	—
Resistance bias	−0.373	−0.435	−0.360	−0.368	0.563	0.897	—	—
Behavioral intention	0.724	0.750	0.782	0.802	−0.369	−0.394	0.971	—
Use behavior	0.600	0.644	0.611	0.630	−0.326	−0.424	0.785	0.777

^a^The items on the diagonal represent the square root of the average variance extracted value; the off-diagonal elements are correlation estimates.

^b^Not applicable.

### Structural Model Analysis

The fitness of the model was evaluated with the following criteria: (1) the standardized root mean square residual (0.0317) had to be smaller than 0.08, (2) the comparative fit index (0.968) had to be greater than 0.90, and (3) the root mean square error of approximation (0.056) had to also be smaller than 0.08. As shown in Table 5, most of the indicators satisfied the criteria and the combination rule [44], which demonstrated that the hypothesized model was a good fit to the data.

**Figure 2:**
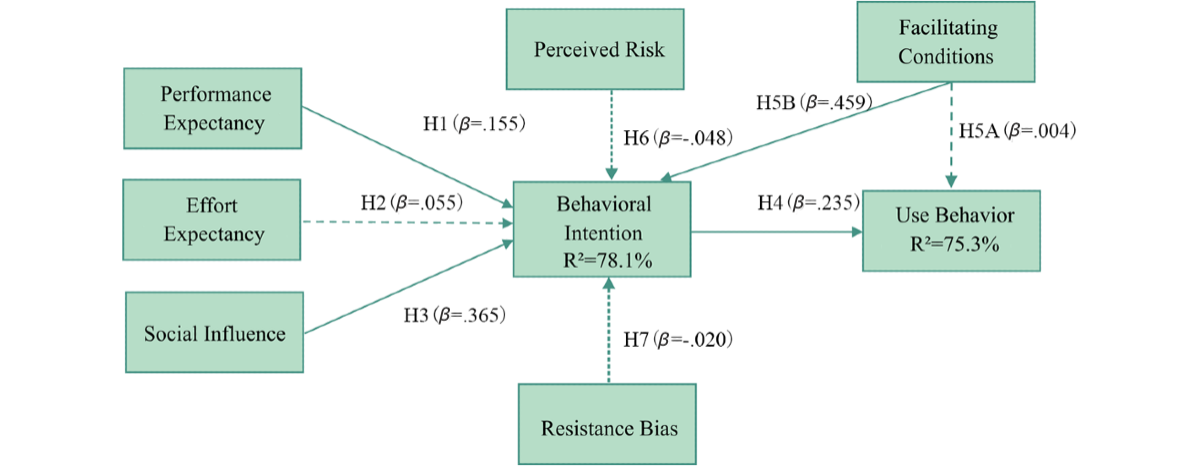
Estimates from the regression analysis. H: hypothesis.

Figure 2 shows a graphic description of the regression analysis, and Table 6 shows the numerical results of the path coefficient analysis. Standardized factor loadings (ie, *β*) were the standardized regression coefficients for each proposed path, which indicated the relationships among the variables in this study. *P* values were the parameters used to determine the results of the hypothesis test [45]. Behavioral intention was positively affected by performance expectancy (*β*=.155; *P*=.014), social influence (*β*=.365; *P*<.001), and facilitating conditions (*β*=.459; *P*<.001), and use behavior was significantly affected by behavioral intention (*β*=.235; *P*<.001). Effort expectancy (*β*=.055; *P*=.450), perceived risk (*β*=−.048; *P*=.348), and resistance bias (*β*=−.020; *P*=.634) did not significantly affect behavioral intention, and facilitating conditions (*β*=.004; *P*=.845) did not significantly affect use behavior. Behavioral intention also moderated the effects that social influence (*P*=.001) and facilitating conditions (*P*<.001) had on use behavior. *R*^2^ values were calculated to assess the validity of the research model; 78.1% of the effects of behavioral intention could be explained by perceived risk, effort expectancy, social influence, facilitating conditions, performance expectancy, and resistance bias, and 75.3% of the effects of use behavior could be explained by behavioral intention and facilitating conditions (Figure 2).

**Table 5:** Research model fit.

Model fit indices	Criteria	Research model fit values
Chi-square value	The smaller the better	692.543
Degrees of freedom	The larger the better	354
Normed chi-square value (*χ*^2^/df)	1<*χ*^2^/df<3	1.956
Root mean square error of approximation	<0.08	0.056
Standardized root mean square residual	<0.08	0.0317
Comparative fit index	>0.9	0.968
Goodness of fit index	>0.85	0.859
Adjusted goodness of fit index	>0.8	0.827

**Table 6:** Summary of support for the hypotheses.

Variables and hypotheses	Standardized factor loadings (SE)	Critical ratio (T value)	*P* value	Results
Hypothesis 1: Behavioral intention is affected by performance expectancy.	0.155 (0.063)	2.464	.01	Supported
Hypothesis 2: Behavioral intention is affected by effort expectancy.	0.055 (0.073)	0.755	.45	Not supported
Hypothesis 3: Behavioral intention is affected by social influence.	0.365 (0.076)	4.795	<.001	Supported
Hypothesis 4: Use behavior is affected by behavioral intention.	0.235 (0.024)	9.778	<.001	Supported
Hypothesis 5A: Use behavior is affected by facilitating conditions.	0.004 (0.022)	0.195	.85	Not supported
Hypothesis 5B: Behavioral intention is affected by facilitating conditions.	0.459 (0.061)	7.561	<.001	Supported
Hypothesis 6: Behavioral intention is affected by perceived risk.	−0.048 (0.051)	−0.939	.35	Not supported
Hypothesis 7: Behavioral intention is affected by resistance bias.	−0.020 (0.042)	−0.476	.63	Not supported

## Discussion

### Principal Findings

In this study, we investigated factors that may affect the adoption of an AI-assisted technology for developing treatment plans for cancer radiation therapy patients. The constructs of the research model were developed based on the UTAUT model, and perceived risk and resistance bias were added as potential barriers to new technology adoption. There were 3 principal findings, as follows: (1) the physicians’ overall perceptions of the AI-assisted technology for radiation contouring were high; (2) social influence and facilitating conditions played a much more important role in AI adoption among radiation oncologists than performance expectancy, but effort expectancy was not significantly related to behavioral intention (*P*=.45); and (3) perceived risk (*P*=.35) and resistance bias (*P*=.63) were not significantly related to behavioral intention.

### The Physicians’ Overall Perceptions of the AI-Assisted Technology for Radiation Contouring Were High

Participants had high behavioral intentions (score: mean 5.484 out of 7.0) to use the new AI-assisted contouring technology; 90.9% (279/307) of the total sample gave scores of 4 or above, meaning that they were planning to use the system within 1 year. This result nearly matched those of a survey conducted in 2019, in which 84.62% of the participating medical departments planned to try the AI-assisted contouring system; 10.26% of these departments were in the process of purchasing the system, and 46.15% had purchased plans [46].

Participants’ performance expectancy and effort expectancy were high, as they believed that the AI technology could improve their work performance and efficiency. This is similar to the perceptions of Canadian radiation oncologists, who believe that AI could save time and deliver better results to benefit patients [47].

### The Relationship Among Performance Expectancy, Social Influence, and Facilitating Conditions Affected Behavioral Intention

Many studies have proven that performance expectancy, effort expectancy, social influence, and facilitating conditions have positive effects on use behavior [8,11,20,48]. For example, in a study of health care robot adoption, these four constructs significantly predicted the usage and uptake of robots [30]. In our study, performance expectancy (*P*=.01), social influence (*P*<.001), and facilitating conditions (*P*<.001) were significantly related to behavioral intention, while effort expectancy did not significantly affect behavioral intention (*P*=.45)*.* Social influence and facilitating conditions affected behavioral intention much more strongly than performance expectancy. These results indicate that in China, when physicians encounter AI-assisted technology for treatment plans, their opinions are more likely to be driven by people with which they have a close relationship (ie, the items of the social influence construct), such as friends and colleagues, department heads, tutors, superiors, and hospital leaders. This phenomenon reflects the culture of vertical collectivism, which is aligned with the ideology of “utilitarian Guanxi”—a Chinese philosophy that combines the pursuit of profit with objective goals [49]. Further, social propaganda, such as news stories about successfully using AI technology from professionals in the health care industry, likely influences physicians’ perceptions toward using such technology.

Facilitating conditions did not have a direct effect on use behavior. However, this construct was the most important factor that affected behavioral intention. As shown by our results, behavioral intention moderates the effect that facilitating conditions have on use behavior (*P*<.001), indicating that providing support, such as support from the information technology team in a hospital, and having the knowledge and skills needed for using new technology were necessarily important and influenced radiation oncologists’ use intentions. On the other hand, most researchers that use the UTAUT model agree that effort expectancy has a significant effect on behavioral intention. However, there have been some exceptions. For example, perceived ease of use did not have an effect on attitudes related to health care professionals’ decisions to accept telemedicine technology [50]. In our study, effort expectancy did not affect use intention. In China, physicians in level 3 public hospitals (the highest standard in China) usually have heavy workloads. For example, at Sun Yat-sen University Cancer Center, the Department of Radiation Oncology works on approximately 1200 cases each day. The participants claimed that they did not mind putting in more effort and spending more time on learning about the new technology in order to later reduce their workloads. Determining whether this approach reduces or redistributes workloads is an area worthy of future investigation. Additionally, the AI technology was developed based on participants’ previously used software. As a result, they found it easier to gain fluency with the system.

### Perceived Risk and Resistance Bias Were Not Significantly Related to Behavioral Intention

In most research on new technology adoption, perceived risk has negatively affected the public’s behavioral intentions [11,29]. However, in our study, perceived risk did not affect radiation oncologists’ behavioral intentions. Even though they perceived risks (score: mean 4.418 out of 7.0) such as the possibility of being misled with inaccurate contouring and privacy issues, oncologists still had the intention to use the system, as did others (colleagues and superiors). The main reason for this was that it could effectively reduce their heavy workloads. In interviews with some of the participants, physicians explained that they could manually fix the errors in the contouring area because the amount of patient case data has increased and machine learning has made the AI-assisted contouring system more and more accurate; therefore, more time will be saved in the future.

As discussed in a study by Ye et al [10], resistance bias has had a negative impact on the technological acceptance of AI ophthalmic devices. However, different from the general public’s perceptions, the low score for resistance bias (score: mean 3.528 out of 7.0) in our study showed that radiation oncologists were not likely to reject AI-assisted contouring technology, regardless of their unfamiliarity with the technology, their past experiences with the failure of new technology products, or the possibility of AI replacing and displacing jobs.

Our study demonstrated that resistance to using an AI-based contouring system to improve patient outcomes and work efficiency was low. There was little to no apprehension among users to learn about and implement this new technology. In previous studies of technology acceptance, resistance bias was observed among medical staff when the benefits of technology were not clear or immediate [36,51,52]. In past research on health care robot adoption, resistance bias did not have a direct negative effect on medical staff’s behavioral intentions, as the health care robots were convenient to use and provided significantly better results than those provided by traditional methods [30]. In our study, the AI platform was developed by using the previous software that the oncologists used for work. This resulted in immediate benefits for improving contouring results and saving time. Therefore, negative perceptions surrounding new technologies with clear and immediate benefits, particularly fear and apprehension toward using such technologies (regardless of the degree of these perceptions), may not be prevalent in China. On the other hand, the AI contouring system in our study is used as an assistance tool for providing image contouring results as part of the process for developing a radiation therapy plan. In our case, oncologists still have to work on modifying the contouring areas and deciding on the amounts of radiation for each area. Wong et al [47] conducted a study among Canadian radiation oncologists that showed similar results; 77% of the participants agreed that human oversight was necessary for optimizing the planning process and delivering better quality care.

In China, using AI-assisted technology to relieve physicians’ workloads will be a major trend in the future, as such technology tries to fill the gap between the public’s demands and the limited amount of medical resources. Chinese hospitals have the need and motivation to adopt AI to improve efficiency, solve the health care professional shortage problem, and reduce patient waiting times. At Sun Yat-sen University Cancer Center, a breakthrough point for the AI product was the potential research opportunities with the AI development company for improving treatment planning accuracy, as the AI product not only helps oncologists improve their work efficiency but also serves as a valuable tool for scientific research.

Hospitals in China, like those in other Asian countries such as India, South Korea, and Thailand, have also proudly indicated their usage and acceptance of AI technology in their marketing campaigns, which have expounded to patients how much their medical services are improved by such technologies [18]. However, anecdotal reports on AI products in cancer care developed by IBM Corporation, one of the largest AI technology providers in the industry, have shown that the company has had trouble with finding customers in the United States [53] and that technology resistance exists among oncologists who claim that they trust their own judgment over that of software [18]. We suggest that cultural differences may result in different perceptions on AI adoption in Western and Asian countries. Asian physicians may be more likely to accept the idea that AI technology is an assistant that performs certain routine tasks and more likely to reject AI doctors. In this case, the perception is that the AI system will not displace or replace the clinical practitioner; instead, the system will get rid of routine tasks and thus allow for more time to focus on patient care.

### Implications for Practice

This study provides 3 suggestions for health care AI developers and hospitals that are planning to introduce AI for clinical decision-making. First, when a hospital introduces a new AI-assisted technology for clinical decision-making in China, the leadership should consider a top-down management process. Hospital leaders and department heads should demonstrate how a new technology works, advocate for the new technology, and encourage subordinate physicians to use the new technology. The hospital could also share AI success stories to influence public opinions on the use of the new technology and highlight the benefits of the system. At Sun Yat-sen University Cancer Center, breakthrough news stories about AI clinical research have been shown not only on social media platforms and local news programs but also at special events such as AI versus human competitions, which have been held at a number of academic conferences, to prove the accuracy of their AI system to both the public and experts. Second, the user interface of the new AI-assisted system should be as consistent as possible with the previously used interface to reduce the time required for users to adapt to the new technology. The hospital information technology center should also work closely with AI technology companies to provide technical support and data security for the use of the new AI platform. Third, per the lesson learned from IBM Corporation’s failure in the United States, AI technology development (ie, progressing from perceptual intelligence to cognitive intelligence) should be a step-by-step process. IBM Corporation’s oncology expert advisor project used natural language processing to provide treatment recommendations. However, the ambitions of such project were, in hindsight, a step too far. Instead of installing an AI doctor, Chinese hospitals first created an AI technology to act as an assistant tool for medical imaging to free physicians from routine work. When a clear application scenario for medical AI is imagined and created, people are more likely to realize the value of such technology.

### Limitations and Future Research

This study has some limitations. Our results might be biased due to the age of participants, as 87.6% (269/307) of the participants were aged under 40 years. The majority of the participants (176/307, 57.3%) were also from Sun Yat-sen University Cancer Center, where medical capacity is high. Additionally, there might be elements of sampling bias, as convenience sampling was used. Further research needs to be carried out among medical professionals in other countries for comparisons of AI technology acceptance in health care services in different cultural contexts.

### Conclusion

In this study, technology resistance among Chinese radiation oncologists was low and not related to behavioral intention. Negative perceptions of new technologies with clear and immediate benefits, such as fear and apprehension toward using such technologies (regardless of the degree of these perceptions), may not be prevalent in China. Further, not all of the factors in the Venkatesh UTAUT model applied to AI technology adoption among physicians in a Chinese context. Oncologists’ opinions are more likely to be driven by people with which they have a close relationship (the items of the SI construct), such as friends and colleagues, department heads, tutors, superiors, and hospital leaders, and be based on receiving technology support from information technology departments and companies.

## Abbreviations

AI: artificial intelligence
AVE: average variance extracted
TAM: Technology Acceptance Model
UTAUT: Unified Theory of Acceptance and Use of Technology
